# Maxillary Implant-Supported Overdentures: Mechanical Behavior Comparing Individual Axial and Bar Retention Systems. A Cohort Study of Edentulous Patients

**DOI:** 10.3390/medicina56030139

**Published:** 2020-03-19

**Authors:** José Félix Mañes Ferrer, Lucía Fernández-Estevan, Eduardo Selva-Otaolaurruchi, Carlos Labaig-Rueda, María Fernanda Solá-Ruíz, Rubén Agustín-Panadero

**Affiliations:** Department of Dental Medicine, Faculty of Medicine and Dentistry, University of Valencia, 46010 Valencia, Spain; jose.manes@uv.es (J.F.M.F.); eduardo.j.selva@uv.es (E.S.-O.); carlos.labaig@uv.es (C.L.-R.); m.fernanda.sola@uv.es (M.F.S.-R.); rubenagustinpanadero@gmail.com (R.A.-P.)

**Keywords:** dental implants, overdenture, locator, bar, clinical outcomes

## Abstract

*Background and objectives:* To compare the medium- to long-term mechanical behavior of overdentures with two different retention systems: overdentures with Locator® axial retention, and vertical insertion overdentures with bar retention, used to rehabilitate edentulous maxillar. *Material and Methods:* This prospective study assessed patients presenting complete maxillary edentulism, rehabilitated by means of implant-supported overdentures (*n* = 20), 10 with Locator® axial retention (ODA group) and 10 with overdentures on bars (ODB group). Patients also completed a questionnaire to determine their satisfaction with treatment. *Results:* The mean follow-up time in both groups was 11.4 years, with follow-up times in both groups ranging from 5 to 14 years. The ODA group suffered mechanical complications such as retention loss, need for nylon retention insert changes, resin fracture, and need for relining. In the ODB group, prosthetic dental wear, screw loosening, and complete prosthetic failure were more common. A total of 19 implants failed (23.8%); of these, 11 were in the ODA group (failure rate = 27.5%) and eight in the ODB group (failure rate = 20%). The patient satisfaction questionnaire obtained a mean score of 7.9 out of 10 in the ODA group, and 9.75 in the ODB group. *Conclusions:* in rehabilitations of edentulous maxillar by means of implant-supported overdentures, both the systems assessed were shown to be effective in the medium to long term. Patients expressed a high level of satisfaction with the treatments received.

## 1. Introduction

The general population is aging so that by 2015 around 16% will be aged 65 years or over, in comparison with 9% in 2019 [1]. According to a report by the Population Reference Bureau [2], in 2018, in North America 15%, of the population was aged over 65 years and this percentage is expected to rise to 23% by 2050. The report also predicts that, in Europe, the population aged over 60 years will reach 33.6% by 2050. In light of this increase in life expectancy, dental care needs in this age group are bound to grow. So, it is becoming increasingly necessary to provide reliable and predictable treatments that meet the specific needs of the elderly.

Dental loss has negative effects on essential oral function, as well as the social aspects of dental health. Oral disease and edentulism are common problems, making treatment and its consequences for quality of life a chief concern when assessing patients’ overall health status [3,4]. In the last 30 years, dental implant-based treatments have become a valuable treatment option for completely edentulous patients as an alternative to conventional dentures. The therapeutic options are various, including from rehabilitation involving numbers of implants, to a more minimal option represented by implant-supported overdentures.

The overdenture is defined as a removable dental prosthesis that covers and is supported by one or more remaining natural teeth, the roots of such teeth, and/or dental implants [5]. Treatment by implant-supported overdentures is considered a good therapeutic option when it comes to rehabilitating edentulous patients, a treatment that meets both functional and esthetic demands [6,7,8,9,10,11]. In recent years, various authors [12,13,14,15] have affirmed the high survival rates of implants supporting overdentures, a finding that reinforces the indication for this treatment option in completely edentulous patients. This has also been backed by the McGill and York Consensus Statements, which established the implant-supported overdenture placed between the mental foramina as a more effective and minimal treatment option for the completely edentulous mandible than the conventional denture [16,17]. In the edentulous maxilla, no consensus has been reached as to the minimum number of implants necessary to support an overdenture. 

Overdenture design offers multiple possibilities, including a variety of retention systems. The retention systems employed in vertical insertion overdentures may use magnetic retainers, ball systems, bars, or stud attachments. However, when it comes to assessing the different retention systems available in terms of success rates, ease of maintenance, patient satisfaction and preferences, and cost to the patient, recent literature has failed to reach definitive conclusions as to which is the best retention system for overdentures [18,19,20,21,22,23,24].

The aim of this prospective observational study was to compare the medium- to long-term mechanical behavior of two types of retention systems for implant-supported overdentures: vertical insertion overdenture with axial retention (Locator®) (ODA group) and vertical insertion overdenture with bar retention (ODB group), for rehabilitating the edentulous maxilla.

## 2. Materials and Methods

This prospective observational study investigated patients with complete maxillary edentulism treated by means of implant-supported overdentures. The overdentures were classified according to their support, the ODA group were implant-tissue–supported overdenture and the ODB group were implant-supported overdenture [25], both study groups being implant-retained. Twenty patients were included in the study (*n* = 20), 10 per group. 

**Figure 1:**
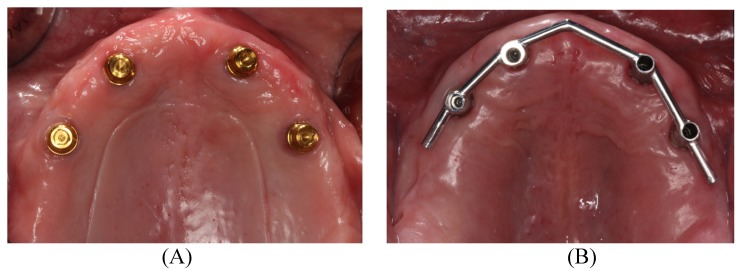
Examples of overdentures: (**A**) Locator® axial attachments (ODA group), and (**B**) bars attachments (overdentures on bars (ODB) group).

All patients received Biomet 3i Certain® internal connection tapered maxillary implants (Zimmer Biomet Dental; Palm Beach Gardens, FL, USA) with a diameter of 4 mm and a length of 10 mm placed by two operators at the clinic run as part of the Masters’ program in Dental Prosthetics at the University of Valencia. Ten patients were rehabilitated with Locator® axial attachments (ODA group), and 10 with overdentures on bars (ODB group) (Figure 1A,B).

The study protocol was approved by the University of Valencia Ethics Committee (registry number 070217; Approved on 23 February 2011) for Research in Humans. All patients gave their informed consent to undergo the treatments described. 

Patients were selected according to the following inclusion criteria: healthy adults (ASA I), complete edentulism in the maxilla with bone availability for direct implant placement in natural maxillary bone without the need for grafts or bone regeneration, subjects not diagnosed with bruxism or TMJ disorders (before the study began), no antecedents of bisphosphonate administration, patients rehabilitated with implant-supported overdentures, patients with detailed medical histories available, and a follow-up period following overdenture placement of at least 5 years, patients who had attended annual dental check-ups for correct maintenance of the prosthetics. As an additional inclusion criterion, all patients were also edentulous in the mandible and rehabilitated by means of implant-supported resin prostheses, overdenture type. 

Exclusion criteria were as follows: patients with natural teeth in the antagonist arch (mandibular); patients rehabilitated with ceramic prostheses in the mandible; patients with incomplete clinical histories, or with follow-up periods of less than 5 years. 

Data drawn from the patients’ clinical histories were reviewed prospectively, their current status assessed, as well as patient satisfaction with treatment. Satisfaction was assessed by means of a survey conducted by telephone or in the clinic. For each patient, mechanical complications, implant and prosthesis survival, follow-up period and patient satisfaction were evaluated.

All data were entered in a Microsoft® Excel spreadsheet. For statistical analysis, descriptive statistics were calculated using the SPSS for Windows® (Statistical Package for the Social Sciences. SPSS Inc., Chicago, IL, USA) for continuous ordinal variables: mean, standard deviation, minimum, maximum, and median, as well as frequencies and percentages for each category. Inferential analysis consisted of survival analysis of any events (prosthesis failure, fracture, etc.) undergone by prostheses following the Kaplan–Meier method. Accumulated survival was calculated and mean survival time, with standard error, and a 95% confidence interval. Differences in survival according to prosthetic type were evaluated by means of the log-rank test. The significance level set for all bivariate analyses was 5% (*p* = 0.05). The sample size (*n* = 20) only permitted a power of 60% to detect a relative risk of RR = 4 in the association of an outcome and a two-level independent factor (such as type of prosthesis) for a 95% confidence interval. 

## 3. Results

The mean age of patients was 72 years; 60% were women and 40% men. All visited the dentist regularly, and maintained oral hygiene, averaging two tooth-brushings per day. 

**Figure 2:**
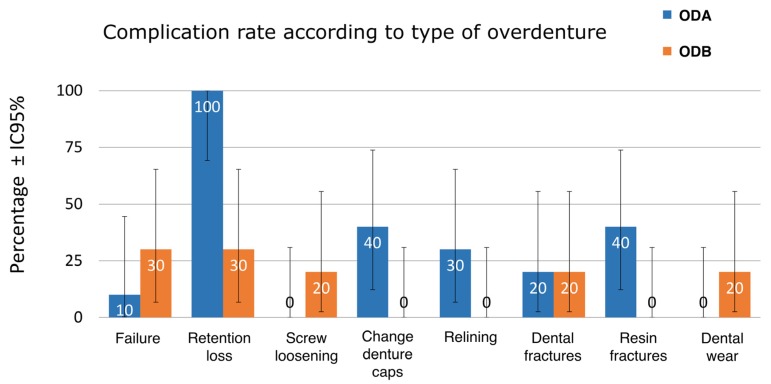
Complication rates according to type of overdenture retention. Vertical insertion overdenture with axial retention (Locator®) (ODA group) and vertical insertion overdenture with bar retention (ODB group).

Taking an overall view of the results, comparing events or complications between the two types of retention (Table 1), the ODA group suffered more problems related to retention loss, retention insert changes, resin fracture, and relining while the ODB group suffered more prosthetic dental wear, screw loosening, and complete prosthetic failure, although differences were nowhere near statistical significance (Figure 2). 

The results obtained for each group were as follows: a total of 19 implant failures (23.8%); of these, 11 were in the ODA group (implant failure rate = 27.5%), and eight in the ODB group (implant failure rate = 20%).

Regarding implant survival, in the ODA group mean follow-up time was 11.4 years, ranging from 5 to 17 years. Of the 40 implants included, 11 were lost in five patients, making a survival rate of 72.5%. Five implants were lost during the first year due to lack of osteointegration, of which three were lost in a single patient (a smoker). Another four implants were lost after four years following overdenture placement, three of these in the same patient, so treatment was regarded as having failed. The other two implants were lost after 11 years. Of the 11 failed implants, eight were replaced successfully in four patients. One patient decided not to have the implants replaced. 

So, for the ODA group, prosthetic survival after 11 years was 90%, as 10 overdentures continued to function adequately, while one failed due to implant loss after four years. 

For the ODB group, the mean follow-up time was 11.4 years, ranging from 5 to 14 years. 

Of the 40 implants paced, eight were lost in three patients, making an overall survival rate of 80%. During the first year after overdenture placement, one patient lost three implants and another lost four, the latter leading to treatment failure. One patient lost one implant after four years, resolved by shortening the retention bar, after which the overdenture continued in use supported by three implants for a further six years. During the 11.4-year follow-up, the prosthetic survival rate for this group was 70%, as three of the 10 prostheses failed. Two failed within the first year due to implant loss. One case lost one implant after four years but the prosthesis was left in place, supported by three implants for a further four years; after 10 years, both the lost implant and overdenture were replaced, fabricating a new bar and a new overdenture. 

**Figure 3:**
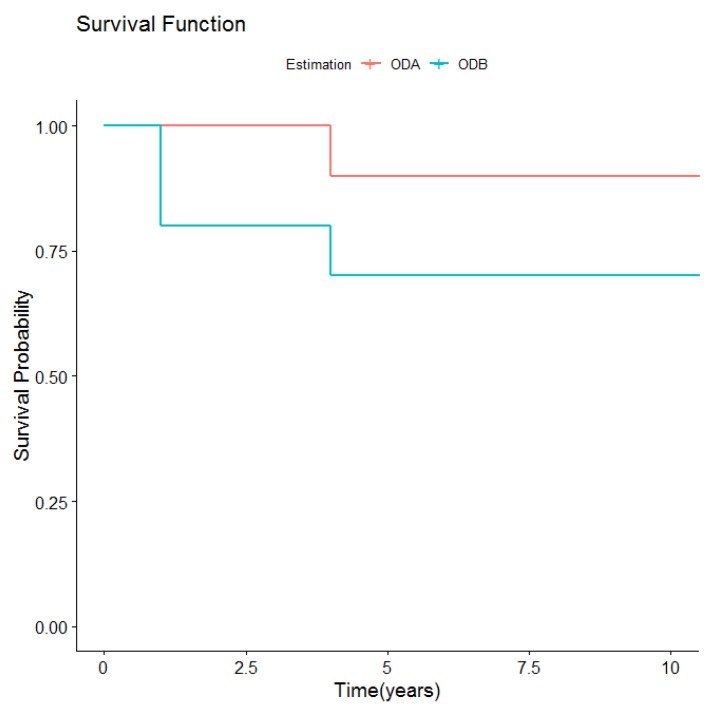
Overall prosthetic survival according to retention type.

However, there were no significant differences in prosthetic survival curves (Table 2 and Figure 3) between the two types of overdenture retention. 

Analyzing mechanical complications in the two groups, different events were observed. 

**Figure 4:**
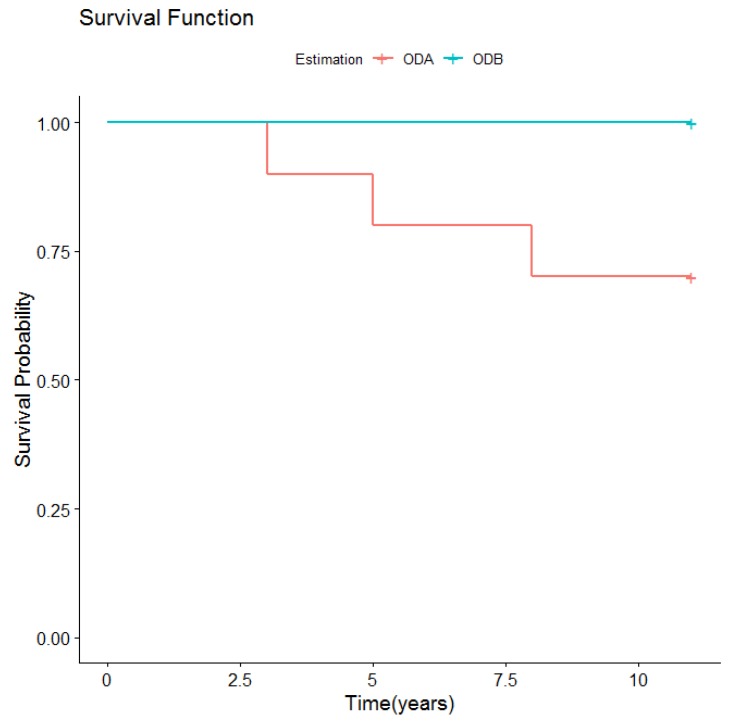
Need for relining according to type of overdenture retention.

In the ODA group, the most frequent complication was retention loss of the Locator system due to progressive wear to the abutments. During the first five years, the decrease in retention was resolved by changing the nylon retention inserts in a mean of 35% of patients with one change every three years. The most common cause of retention insert changes was wear or deactivation (71.7%), followed by insufficient retention (30.2%). Nevertheless, after five years, changes in inserts became more frequent and less effective due to abutment wear or breakage. Five patients were advised to replace abutments, three patients after 10 years and two after six years. One patient did not do so until seven years later, 11 years after treatment. In two patients, abutments had to be changed due to their fracture, one after five years, another after eight years. Another frequent mechanical complication was the need to change denture caps (the system housing the nylon retention inserts). Six caps were changed due to their loss in four patients: one after three years, three after seven years, and two after eight years. Regarding the number of relinings carried out in this group, three patients needed four relinings over the period described: one after three years, one after two years, one after five years, and another after eight years of overdenture use (Figure 4). 

**Figure 5:**
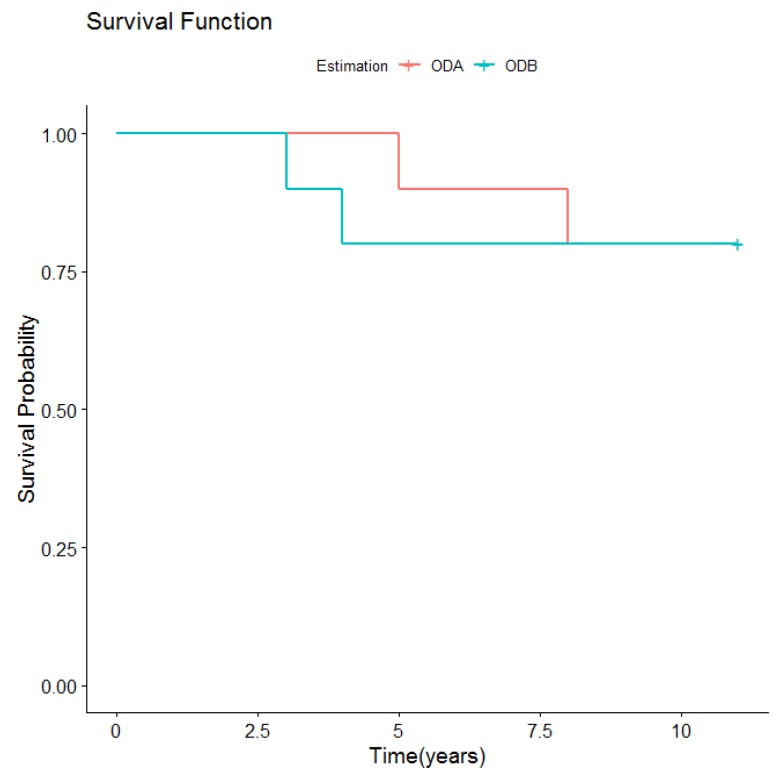
Prosthetic dental fractures according to overdenture retention type.

Lastly, prosthetic dental fractures also occurred in this group. Three prosthetic dental fractures were produced in two patients. One of these was due to a fall, eight years after overdenture placement; in another patient dental fractures occurred twice, the first time after five years and the second time after eight years. In all cases, the fractured teeth were replaced and the overdenture continued to function normally (Figure 5).

**Figure 6:**
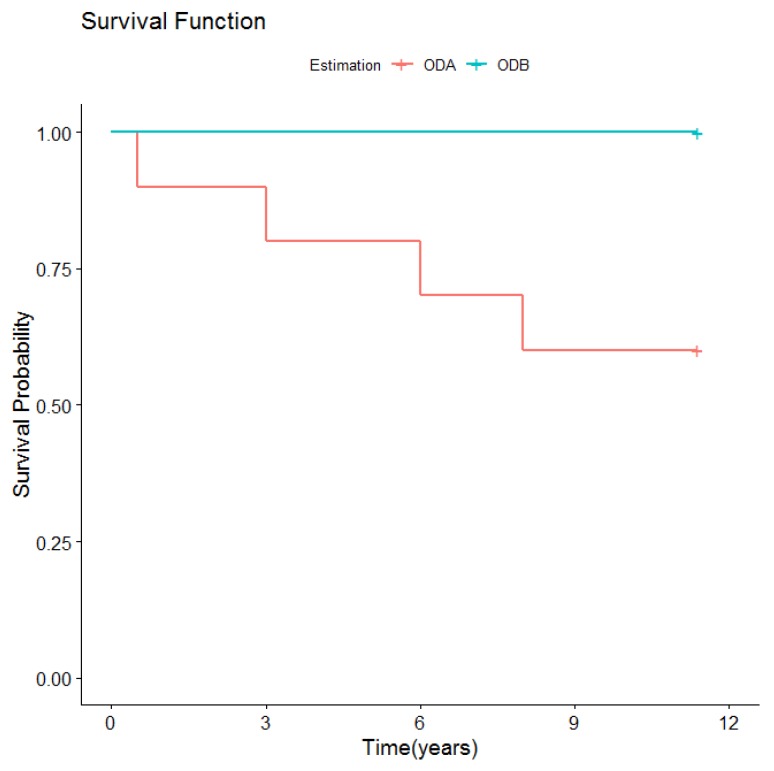
Occurrence of resin fractures according to overdenture retention type.

Another type of fracture affected overdenture resin. Four overdentures belonging to four patients had to be sent for repair. One after six months, which was reinforced with a metal mesh; in the other three, fractures occurred after three, six, and eight years. All continued to function adequately after repair (Figure 6). 

**Figure 7:**
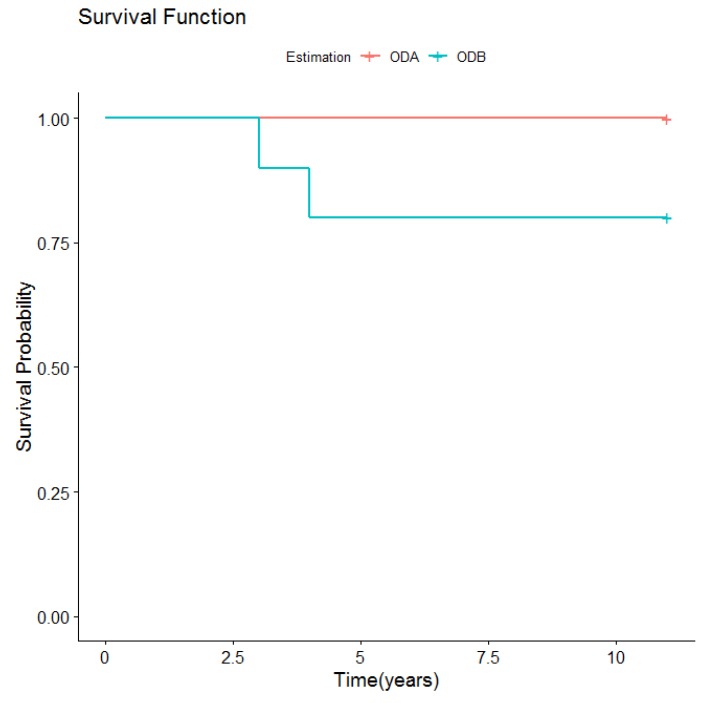
Retention screw loosening/loss according to type of overdenture retention.

The most common mechanical complication in the ODB group was loss of retention due to screw loosening or fracture of the retention rider clips, which occurred three times in three patients, after 6, 9, and 10 years. Another complication was loosening or loss of the bars’ retention screws; one patient lost three screws after three years, which were replaced with new ones. In another patient, screw loosening occurred after four years (Figure 7).

**Figure 8:**
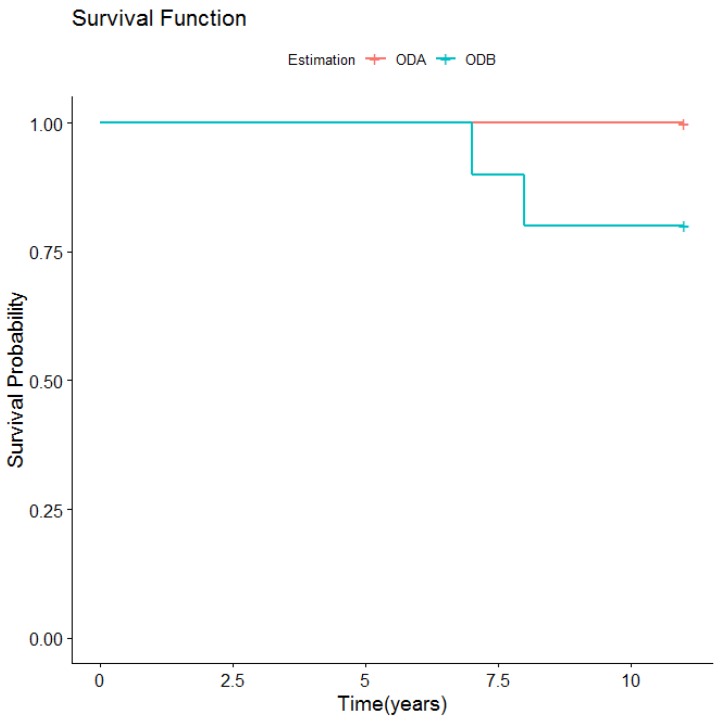
Dental wear according to type of overdenture retention.

Two prosthetic dental fractures were observed, one after three years’ and another after four years’ use. Regarding dental wear, teeth had to be replaced in two patients, after seven years in the first case, and eight years in the second. Wear was also observed in another two patients after 6 and 10 years, respectively, but in these cases the teeth were not replaced (Figure 8). 

Comparing the incidence of mechanical complications between the two groups, it should be noted that the ODA (implant-tissue–supported overdenture) group presented more need for relining due to the system’s resilience in comparison with the OBD group (implant-supported overdenture), which did not need this procedure (*p* = 0.067).

Overdentures in the ODA group suffered resin fractures, while the ODB group did not present this complication (*p* = 0.029).

At the same time, the ODB group presented a higher number of mechanical events affecting the prosthetic teeth (wear or fracture), as well as more complications derived from loosening or loss of the retention screws between bar and implants, although the differences between the groups did not reach statistical significance. 

**Figure 9:**
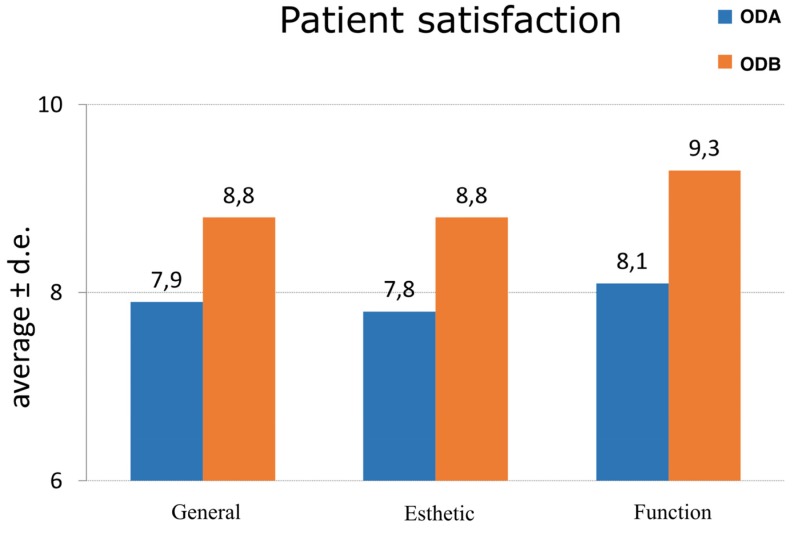
Results of patient satisfaction survey according to overdenture retention type (average values with standard deviation (d.e.).

Regarding the results of the Oral Satisfaction Survey, in the ODA group patients awarded their overdentures an average score of 7.9 out of 10. Satisfaction with esthetics was 7.8, patients complaining of the color of teeth, a lack of natural appearance, and excessive vestibular ‘skirt’. For function, the score rose to 8.1, with most patients reporting improvement in eating, speaking, etc. In the ODB group, the survey obtained higher scores, for overall satisfaction (average of 8.75 out of 10), esthetics (8.75), and function (9.25) (Figure 9).

## 4. Discussion

Since the introduction of osteointegrated implants, implant-supported overdentures have become a good treatment option for the completely edentulous patient. The indications for rehabilitation by overdentures were established in the 1990s [26].

The implant survival rate (ISR) in this type of treatment has been extensively documented [27,28,29,30,31,32], with rates varying from 91.9% to 100%. In comparison with these rates, the present work obtained relatively low ISRs in both the ODA and ODB groups. These lower rates may be due to the fact that the study had a minimum follow-up period of five years, while the studies cited above had follow-up periods ranging from one to five years, only one study by Slot et al. having a five-year follow-up [32]. In the present case, the ISR in the ODA group was 72.5%, due to implant loss in the first year caused by a lack of osteointegration, of which three implants were lost in one patient (a smoker), an observation that concurs with research by Vercruyssen and Balaguer [28,33], who found that ISRs were lower among smokers. Another four implants were lost four years after overdenture placement, three of these in the same patient leading to overdenture treatment failure. A further two implants were lost, one of them after 11 years and the other after 16 years. In a literature review conducted by Raghobear et al. [34], ISR varied between 72.4% and 100%, making an average survival rate for non-splinted implants of 88.9%; the mean follow-up period in the studies selected for review was 31.5 months. 

In the present work, the ISR was 80% in the ODB group, with a mean follow-up of 11.4 years, with a patient age range of 5-14 years. Comparing these results with three recent literature reviews, the present results are clearly worse. In one review by Leao et al. [22], mean follow-up of the studies analyzed was five years ranging from nine months to 10 years. The review obtained a mean ISR of 93.3%. Raghoebar et al. [34] report ISRs for bar-retained overdentures varying from 97% to 98.1% with a mean follow-up of 2.6 years ranging from 1 to 10 years. Lastly, the review by Slot et al. [15] reported ISRs ranging between 96.3% and 98.2% in relation to the number of splinted implants used (four or six). The average follow-up of the studies reviewed was four years, ranging from 1 to 15 years. However, analyzing those studies with follow-up periods of over eight years, as in the present work, the ISRs obtained were fairly similar to the present study [35,36,37,38]. Slot et al. reported ISRs varying from 76% to 86.9%, when follow-ups were longer, which suggests that the longer implant-supported overdentures remain in use, the lower the ISR. Nevertheless, of the 40 implants placed in the present study, eight were lost in three patients during the first year after placement of the overdenture, with one patient losing three implants and another losing four, which led to treatment failure. The remaining case lost one implant after four years, so the bar was shortened and the overdenture remained supported by three implants for a further six years. 

Regarding prosthesis survival rates (PSR), for bar-retained overdentures (ODB group) this was 70% as three overdentures out of 10 failed, a similar result to Visser et al., whose study had a similar follow-up period (over five years) to the present work [35,37,38], while other studies obtained much better results [36,39,40], also with follow-up periods of over five years. 

The literature makes it quite clear that implant-supported overdentures frequently suffer mechanical complications. In the present work, it may be concluded that the ODA group presented a complication-free period of time preceding retention insert changes or resin fractures that was significantly shorter than the ODB group. A strong tendency for relining was also observed. In the ODB group, prosthetic dental wear and retention screw loosening were more frequent, although these differences did not reach statistical significance. 

As early as 1996, Jemt et al. [41] reported that the incidence of mechanical complications suffered by overdentures was 77.9%, a percentage backed in further studies by Visser and Slot [15,16,17,18,19,20,21,22,23,24,25,26,27,28,29,30,31,32,33,34,35,36,37,38]. The most frequent mechanical complications were: retention loss in retention systems, wear and/or fracture of the teeth or the prosthesis, loss of adaptation of the prosthesis’s base, screw loosening or fracture, or abutment loss. In the present study, the most frequent complication in the ODA group was Locator® system retention loss, whether due to wear to the nylon retention inserts or to the abutments, leading to a need for relining; another common problem was dental and resin fracture. The data obtained agree with Wang et al. [29] who found the most common mechanical complication in Locator-retained overdentures to be retention loss, requiring replacement retention inserts in 21 cases out of a total of 26 patients. Similar data were obtained in studies by Lian, Marinis, and Güedat, who reported the most frequent mechanical complication to be retention loss from the nylon inserts [42,43,44]. Lou reported better results in similar work to the present study comparing axial Locator-retained, bar-retained, and telescopic crown-retained overdentures [27]. In this work, the Locator-retained group did not suffer any notable mechanical complications, although the follow-up period was only three years. Nevertheless, all the other authors cited above reported the need for relining, dental and resin fractures, as frequently occurring mechanical complications. 

Regarding the mechanical complications suffered by bar-retained overdentures, a recent systematic review [22] compared differences between overdenture attachments, with and without splinting, describing the following: abutment or abutment screw loosening, relining, dental or resin fractures, and retention clip breakage, the same complications as observed in the present study. Analyzing the incidence of mechanical complications in the present work, the results were similar to Slot et al. [32], who found that with bar types of overdenture retention, the most common mechanical event was the fracture of prosthetic teeth or resin [45,46].

As for patient satisfaction with treatment, the present study obtained an overall result of 8 out of 10, indicating that implant-supported overdentures met patients’ expectations and may be considered an adequate treatment for maxillary edentulism. The results are similar to those of other authors for this type of treatment, who all obtained scores of over eight on a scale from 0 to 10 [32,38,42,43,47]. 

## 5. Conclusions

Within the limitations of the present study, in particular its small sample size, it may be concluded that rehabilitation of edentulous maxillar by means of the treatments described are effective in the medium to long term, and patients expressed high levels of satisfaction with the overdentures. Nevertheless, it is clear that mechanical complications are frequent and their incidence increases with time. In terms of mechanical complications, the ODA group obtained better results than the ODB group, the latter presenting problems that were more complicated to resolve. 

## Figures and Tables

**Table 1:** Data compiled after clinical follow-up of both overdenture retention types.

Prostheses	Implant Survival (%)	Prosthesis Survival (%)	Mechanical Complications						
ODA Prostheses N = 10 Number of implants supporting each prosthesis: 4 Total number of implants N=40	Of 40 implants, 11 failed: 5 in the first year, four in the 4th year, and 2 in the 11th year.	90%: of 10 prostheses, 1 failed after 4 years.	100% Retention loss (changing inserts every 3 years) Change of abutment: 5 due to wear (after 6, 10 and 11 years) 2 due to fracture (after 5 and 8 years)		40% Change of inserts: 6 changes (one after 3 years, three after 7 years, two after 8 years)	30% Relining: 3 patients (after 2,3, 5 and 8 years)	20% Dental fracture: 3 fractures (1 patient with 2 fractures after 5 and 8 years, the other after 8 years)	40% Resin fracture: 4 fractures (after 6 months, 3, 6 and 8 years)	
**ODB** Prostheses N = 10; Number of implants supporting each prosthesis: 4; Total number of implants N = 40	Of 40 implants, 8 failed: 7 in the first year, and 1 during the 4th year.	70%: of 10 prostheses, 2 failed within the first year and one after 4 years.	30% Retention loss of rider clips: 3 times in 3 (after 6, 9 and 10 years)	20% Screw loosening or fracture: 4 in 2 patients (after 3 and 4 years)			20% Dental fractures: 2 fractures in two patients (after 3 and 4 years)		20% Dental wear: 2 patients (teeth were changed in one patient after 7 years, and another after 8 years)

**Table 2:** Log-rank test results: homogeneity in relation to survival of prosthetic failure events according to the type of overdenture.

Survival according to mechanical behavior	***p*-Value**
**Retention type**	0.251
